# *Notes from the Field:* Emergency Visits for Complications of Injecting Transmucosal Buprenorphine Products — United States, 2016–2018

**DOI:** 10.15585/mmwr.mm6932a5

**Published:** 2020-08-14

**Authors:** Sukarma Tanwar, Andrew I. Geller, Maribeth C. Lovegrove, Daniel S. Budnitz

**Affiliations:** ^1^Epidemic Intelligence Service, CDC; ^2^Division of Healthcare Quality Promotion, National Center for Emerging and Zoonotic Infectious Diseases, CDC.

The opioid partial agonist buprenorphine is a critical component of medication-assisted treatment for opioid use disorder and is associated with improved treatment adherence and decreased illicit opioid use (*1*). Combination buprenorphine/naloxone transmucosal products are designed to deter injection owing to the opioid-antagonist actions of naloxone and can reduce the desired effects and precipitate rapid withdrawal when these products are administered intravenously; nonetheless, injection of transmucosal buprenorphine/naloxone has been reported (*2*,*3*). During 2016–2017, 14.6% of approximately 127,000 emergency department (ED) visits for nonmedical use* of prescription opioids involved buprenorphine products, commonly for injection-related complications (*4*). ED visits for nonmedical use of buprenorphine involved less severe overdose morbidity (e.g., unresponsiveness or cardiorespiratory failure) than did those involving other opioids (*4*). Complications of injecting transmucosal buprenorphine products represent a potentially preventable source of morbidity from nonmedical use of buprenorphine. Further description of complications related to buprenorphine injection can help prevent these complications while preserving access to this effective therapy for opioid use disorder.

During 2016–2018, among ED visits tracked by the National Electronic Injury Surveillance System-Cooperative Adverse Drug Event Surveillance project, a nationally representative active public health surveillance system (*5*), 598 cases of nonmedical injection of prescription opioids were identified by record review. CDC used these cases to derive an estimate that an average of 47,437 (95% confidence interval [CI] = 27,004–67,871) ED visits for nonmedical injection of prescription opioids occurred in the United States annually. Of these ED visits involving nonmedical injection of prescription opioids, approximately one third (34.2%; 95% CI = 19.3%–56.1%) involved transmucosal buprenorphine products.

Among estimated ED visits for nonmedical injection of transmucosal buprenorphine, mean patient age was 33 years (range = 20–56 years), and two thirds (66.0%; 95% CI = 60.9%–71.0%) of patients were men. ED visits for nonmedical injection of transmucosal buprenorphine usually involved a transmucosal buprenorphine/naloxone combination product (85.4% [95% CI = 76.3%–94.5%] of estimated visits). An estimated two thirds (66.0%; 95% CI = 43.0%–89.0%) of buprenorphine nonmedical injection visits resulted in the patient being treated and released or leaving against medical advice. Concurrent use of nonpharmaceutical substances (e.g., heroin, cocaine) was documented in approximately one third (31.6%; 95% CI = 21.7%–41.6%) of estimated visits for nonmedical injection of buprenorphine.

Injection-specific complications were documented in an estimated two thirds (67.2%; 95% CI = 53.7%–80.6%) of buprenorphine nonmedical injection ED visits. Among 101 ED surveillance cases of visits for buprenorphine nonmedical injection-specific complications, those reported included abscess (37), cellulitis (41), infective endocarditis (two), sepsis (two), septic arthritis (two), unspecified injection-site infections (e.g., “hand infection” not further specified) (three), and noninfectious injection-specific complications (e.g., injection site thrombosis/ischemia) (14). The national estimates likely represent an undercount of the true number of visits for injection-related complications because patients might not disclose injections, and secondary chronic infections (e.g., human immunodeficiency virus or hepatitis C) might not be identified.

Buprenorphine treatment is an important component of the public health response to the opioid overdose epidemic. Patients evaluated in EDs and other settings with injection-related complications might be referred to syringe services programs, where available, and educated on infection prevention practices (*6*). Linking these patients to care for underlying substance use disorders and recovery support services might improve recovery rates. Counseling on risks of injecting buprenorphine could be incorporated into patient education regarding medication-assisted treatment and might reduce the frequency of injection complications.

## Footnotes

* Nonmedical use included abuse (clinician diagnosis of abuse or documentation of recreational use), therapeutic misuse (documented therapeutic intent, but not used as directed; e.g., taking buprenorphine to self-treat withdrawal symptoms), or overdoses without documentation of therapeutic intent, self-harm, abuse, or misuse (e.g., patients who have documented overdoses but are unable or unwilling to describe the event).
